# The Etiology of Alopecia Areata

**Published:** 1904-03-19

**Authors:** 


### THE ETIOLOGY OF ALOPECIA AREATA.

Dr. Arthur Whitfield [1] has observed four cases in which eye strain was associated with alopecia areata. A woman had a large bald patch on the vertex which had defied all forms of local treatment. Headache was a marked symptom. She had an error of refraction for which she had worn glasses. After her eyes had been examined and suitable lenses prescribed, the hair began to grow, and the affected area was thickly covered in six weeks' time. Another patient had a patch of alopecia over her left ear. She had headache and well-marked hypermetropia, which was corrected by lenses. Her hair grew rapidly, but a year later the headache returned in the form of violent attacks and migraine, and another patch of baldness developed. Two students suffering from uncorrected myopia were cured of alopecia areata and severe headache by wearing suitable spectacles. Dr. Whitfield finds that, although in the majority of cases of alopecia eye strain has nothing to do with the disease, in a certain percentage, chiefly of adult sufferers, the nervous irritation consequent on an undue effort to focus fine objects seems to determine the onset of the symptoms in people whose health is not the most robust.

[1] Lancet, March 5, 1904.

